# Potential Changes in the Eutopic Endometrium in Endometriosis: A Narrative Review

**DOI:** 10.7759/cureus.92932

**Published:** 2025-09-22

**Authors:** Fanourios Makrygiannakis, Thomas Vrekoussis, Aikaterini Berdiaki, Antonis Makrigiannakis

**Affiliations:** 1 Obstetrics and Gynecology, University of Crete, Heraklion, GRC

**Keywords:** endometrial receptivity, endometriosis, epigenetics, eutopic endometrium, progesterone resistance, stress

## Abstract

Endometriosis is a disease characterized by the presence of ectopic endometrium, mainly within the peritoneal cavity, and is associated with the development of inflammation due to cyclic endometrial alterations. The end result of endometriosis is usually infertility, often leading couples to assisted reproduction techniques (ARTs). In the context of infertility, several approaches have been made, aiming to highlight potential pathophysiological mechanisms; it is now accepted that endometriosis may affect both ovarian and endometrial function.

The current review aims to summarize the main alterations reported on potential eutopic endometrium changes in cases of endometriosis, focusing mainly, but not exclusively, on local inflammatory pathways, angiogenesis, and immune alterations that may affect implantation. New evidence implies that, in cases of endometriosis, macrophage and T helper 17 (Th17) immune profiles are rather pro-inflammatory - a finding possibly explaining reduced endometrial receptivity. Concurrently, the hypoxic stress noted in endometriosis has been considered the cause of alterations in angiogenesis, as well as in epigenetic regulation. This, in turn, may lead to changes in endometrial cells’ metabolism. In the same direction, progesterone resistance has been shown as a potential factor of impaired endometrial cell homeostasis. The proposed mechanisms of eutopic endometrial cell alterations may be set as research targets for therapeutic interventions via designing proper clinical trials.

## Introduction and background

Endometriosis is defined as the presentation of ectopic endometrial parenchyma and stroma. The exact incidence of the disease remains unknown. It is estimated that up to 10% of women of reproductive age may suffer from endometriosis. The prevalence seems greater among women with infertility (up to 50%), and among women with chronic pelvic pain (up to 20%) [1].

Several theories have been proposed regarding the pathogenesis of endometriosis. The most accepted so far is the theory of retrograde menstruation [2]. Blood and tissue debris reach the peritoneal cavity via the fallopian tubes during menstruation. Normally, peritoneal clearance via scavenging systems removes the endometrial debris. In the case of women with endometriosis, endometrial cells are resistant to apoptosis, presenting an increased proliferation rate [3]. The consequent establishment of endometriotic foci is characterized by local inflammation, setting up a condition of local stress [4].

Endometriosis is an estrogen-dependent disease. Endometriotic foci produce estrogen and present with enhanced estrogen signaling, with overexpression of estrogen receptors (ERs) and their downstream pathways [5]. This effect is further amplified by progesterone resistance, due to low progesterone receptor (PR) expression [6].

The symptoms vary among patients, ranging from asymptomatic conditions to infertility and chronic pelvic pain. As a condition of chronic stress, endometriosis has been associated with several disease entities, including autoimmune diseases [7] and inflammatory bowel disease [8]. The burden of the disease is also related to mental disorders, establishing a vicious circle that enhances symptoms, with a negative impact on quality of life (QoL) [4]. The impact of endometriosis on QoL is immense; several QoL determinants have been reported to be associated with endometriosis. Different methodologies have been employed in stratifying patients based on QoL determinants, with the most recent being artificial intelligence [9]. Endometriosis management is rather complicated, since not all signs and symptoms are clearly attributed to the disease. In that view, diagnostic laparoscopy was considered the best choice to establish a diagnosis, along with the acquisition of histological verification. However, the current trend does not consider laparoscopy as essential, and supports ultrasound and magnetic resonance imaging as a non-invasive alternative [10]. Recent consensus statements have been published, aiming to provide guidelines for contemporary practice, clarifying issues regarding both diagnosis and treatment [11].

In the case of endometriosis-related infertility, a multimodal approach is suggested. Recently, a change in lifestyle, mainly nutrition, is considered to improve fertility [12]. In this direction, evidence is suggestive of a positive role for myo-inositol and vitamin D in improving ovarian function [13-15], which is widely accepted to be negatively affected by endometriosis [16]. The cornerstone of infertility treatment in endometriosis is the application of assisted reproduction techniques (ARTs), either in the context of fertility preservation prior to the surgical treatment of ovarian endometriosis [17], or as a means to overcome the dysfunctions attributed to endometriosis, mainly in the ovaries and fallopian tubes. The ART approach may involve either fresh or frozen cycles, with good results [18,19], a fact that can justify the consideration of ART as the gold standard approach in the case of endometriosis. However, despite the enthusiasm caused by this approach, skepticism has been expressed about long-term effects on future offspring, namely the risk of minor congenital heart defects related to ART [20].

Initially, the treatment of endometriosis-related infertility focused on ovarian dysfunction, as this was associated with poor oocyte quality [16]. However, taking into consideration that a successful pregnancy is the result of both good-quality embryos and normal endometrial decidualization, over the last decade, the role of the eutopic endometrium in women with endometriosis has also been under investigation. This is based on the concept that endometriotic foci (namely, ectopic endometrium) present several alterations that may also be identified in the endometrium (namely, eutopic endometrium), leading to endometrial dysfunction and, consequently, to infertility. Evidence so far supports the presence of eutopic endometrial alterations that may contribute to impaired implantation in endometriosis.

The aim of this narrative review is to highlight the main changes observed in the eutopic endometrium, in the case of endometriosis.

## Review

Literature search strategy

Literature search was performed on the PubMed database, focusing on relevant keywords with the restriction “title and abstract.” References from all papers retrieved were also analyzed and assessed in terms of relevance. Important references from the full-text papers were also retrieved.

Estrogen dominance

The impact of estrogen levels on endometrial function has been previously reported. Estrogen is the major hormone of the proliferative phase of the menstrual cycle, priming the endometrium for the effect of progesterone [21]. Estrogen regulates the expression of both ERs and PRs, establishing the cellular prerequisites for the window of implantation (WOI) during the mid-secretory phase of the menstrual cycle. Estrogen signaling involves two receptors, ERα and ERβ. Activated ERα effects are exerted via its translocation to the nucleus, where it binds to estrogen response elements, regulating, in turn, an array of estrogen-dependent pathways [22]. Several estrogen receptor 1 (ESR1) polymorphisms, suggestive of hyperactivity, have been reported to be associated with endometriosis [23]. 

Endometriosis is characterized by estrogen dominance. Despite the fact that circulating estrogen levels do not differ between women with and without endometriosis, it is now evident that endometriotic foci, along with the eutopic endometrium of women with endometriosis, are featured by locally elevated estrogen levels. This is attributed to the overexpression of P450 aromatase, which catalyzes the conversion of androstenedione to estrone (E1) [24-26]. E1, a non-potent estrogen, is thereafter locally converted to the potent estradiol (E2) (via 17β-hydroxysteroid dehydrogenase type 1), the latter establishing a hyper-estrogenic environment [27]. An imbalance between 17β-hydroxysteroid dehydrogenase type 1 and 2 has been reported in endometriotic cells, with 17β-hydroxysteroid dehydrogenase type 2 being significantly down-regulated [28,29]. As a result, 17β-hydroxysteroid dehydrogenase type 1 acts without the inhibitory effect of 17β-hydroxysteroid dehydrogenase type 2, leading to locally increased levels of E2. Estrogen levels are inversely associated with the duration of the WOI [30]. Concurrently, an inappropriate elevation of ESR1 during the WOI has been described [31]. Therefore, the hyper-estrogenic state of endometriosis is not only associated with a shorter WOI, but also with deregulated estrogen signaling, facts that seem to contribute to implantation failure.

In the same direction, local inflammation, along with elevated estrogen levels, induces the expression of nitric oxide synthase (NOS), leading to elevated levels of NO [24], a free radical contributing to vasodilation and cytotoxicity/cell apoptosis [24]. Elevated endometrial cell apoptosis could be a contributing factor toward implantation failure. NO upregulates cyclooxygenase-2 (COX-2) expression, which, in turn, catalyzes the conversion of arachidonic acid to prostaglandin-E2 (PGE2) [32], a well-known mediator of inflammation. Subsequently, PGE2 induces aromatase activity, further consolidating a hyper-estrogenic environment [33,34]. Interestingly, estrogen is a positive regulator of COX-2 [35]. Thus, it can be suggested that there is a positive feedback loop that leads to a continuous sustainment of inflammation with elevated estrogen and PGE2 levels (Table 1).

**Table 1:** Main molecular landmarks of estrogen dominance

Estrogen dominance (main molecular landmarks)
Overexpression of P450 aromatase
Imbalance between 17β-hydroxysteroid dehydrogenase type 1 and 2
Inappropriate elevation of estrogen receptor 1 (ESR1) during the window of implantation (WOI)
Overexpression of nitric oxide synthase (NOS)
Elevated cyclooxygenase-2 (COX-2) expression
Elevated prostaglandin-E2 (PGE-2) concentration

All the above, taken together, may lead to the conclusion that, in endometriosis, an interplay between inflammation and locally elevated estrogen concentrations is established. The impaired estrogen regulation, depicted as the hyper-estrogenic environment, may affect endometrial physiology via: (a) the NO-mediated induction of endometrial cell apoptosis, and (b) the deregulation of the WOI.

Progesterone resistance

Progesterone signaling is the cornerstone of endometrial receptivity. Progesterone is the main hormone involved in ovulation, decidualization, implantation, and early embryonic development. Progesterone effects are exerted via its receptors, namely PR-A and PR-B. PR-A and PR-B have been shown to act in different fashions. In mice, PR-A is considered the driver of uterine PR activity, while PR-B is involved in mammary development and embryonic morphogenesis [36,37]. In humans, however, PR-B is considered the major receptor controlling endometrial epithelial cell proliferation and decidualization, being under the repression of PR-A [38]. During the normal menstrual cycle, PR expression is gradually elevated, reaching its maximum exactly before ovulation, and is reduced thereafter [24]. This alteration is essential for the switch from the proliferative to the secretory phase of the endometrium. Progesterone is the main contributor to decidualization, an aseptic, well-regulated inflammatory transformation of the endometrial epithelium and stroma. This is achieved both by inducing progesterone-dependent genes via genomic PR activity, as well as via the non-genomic downstream signaling of the PRs, resulting in the activation of extracellular signal-regulated kinase/mitogen-activated protein kinase (ERK/MAPK) and protein kinase B (AKT) pathways [36,39]. Decidualization involves both morphological and biochemical alterations that establish a receptive but concurrently selective microenvironment with respect to embryonic implantation [40]. At the time of the WOI, progesterone seems to attenuate E2 action via the upregulation of 17β-hydroxysteroid dehydrogenase type 2 expression and the subsequent transformation of E2 to E1, the latter being significantly less bioactive [41]. This, in turn, minimizes the estrogenic effect on the decidua, which is at its utmost receptive capability.

Endometriosis is characterized by progesterone resistance. It has been shown that, in the case of endometriosis, both the eutopic and ectopic endometrium present a PR imbalance: PR-B expression has been reported as significantly decreased, mainly due to epigenetic changes that deactivate the PR-B gene [42,43]. This is in line with previous reports showing that women with endometriosis and infertility present with a lower endometrial PR-B to PR-A expression ratio [44], with PR-B expression being significantly lower compared to infertile, non-endometriotic controls [45]. 

Progesterone resistance leads to pathological alterations in downstream pathways, being directly linked to decidualization and implantation [46]:

(A) AT-rich interacting domain protein 1A (ARID1A) is a chromatin-remodeling complex protein that modulates gene expression by loosening chromatin from corresponding nucleosomes. It has been shown that infertile women diagnosed with endometriosis present a significant decrease in ARID1A expression [47]. Recently, in the case of endometriosis, ARID1A was reported to be positively correlated with PR, being in physical interaction with PR-A [48]. Interestingly, ARID1A was also reported to be of critical importance for uterine gland function in early pregnancy [49]. Thus, downregulation of endometrial ARID1A and infertility could be the result of endometrial progesterone resistance.

(B) The PR-dependent Indian hedgehog pathway (Ihh-COUP-TFII-Wnt4) is important for decidualization and embryonic implantation. All components involved in the Ihh pathway have been reported as downregulated in women with endometriosis [50], via activation of the Kirsten rat sarcoma virus (KRAS) gene and overexpression of sirtuin 1 (SIRT1) and B-cell lymphoma 6 (BCL6) [51].

(C) Heart and neural crest derivatives-expressed protein 2 (Hand2) is a progesterone-dependent inhibitor of ESR1 signaling [52]. During the secretory phase, Hand2 contributes to controlling the estrogenic effects on the decidua. Hand2-knockout mice present ERα upregulation as a result of uncontrolled, elevated fibroblast growth receptor (FGFR) signaling, leading to activation of the ERK pathway [52]. In endometriosis, Hand2 was reported to be downregulated due to increased promoter methylation [53].

(D) Mitogen-induced gene 6 (MIG-6) is a PR-dependent inhibitor of cell proliferation [54]. In women with endometriosis-related infertility, MIG-6 was found to be downregulated [55]. Additionally, a mouse knockout study showed that MIG-6-related infertility was caused by upregulation of ERBB2 [55]. ERBB2 ablation restored fertility in MIG-6 knockout mice [55].

(E) Forkhead box protein O1 (FOXO1) is a nuclear transcription factor that interacts with PR to induce insulin-like growth factor binding protein 1 (IGFBP-1) and prolactin (PRL), both essential components for endometrial stromal decidualization [56]. FOXO1 contributes to the regulation of 507 out of the 3,405 genes involved in decidualization [57,58]. FOXO1 was previously reported to be regulated by the neurogenic locus notch homolog protein (NOTCH) family (ligands JAGGED2/DLL4 and receptors NOTCH1/NOTCH4). Endometrial stromal cells were found to have downregulated NOTCH signaling, exerting a negative impact on FOXO1 and its downstream cascades [59].

Moreover, it was shown that, in endometriosis, the eutopic endometrium exhibits elevated expression of Calpain-7, which in turn induces AKT hydrolysis and consequently AKT phosphorylation. AKT phosphorylation leads to the phosphorylation of FOXO1, excluding FOXO1 from the nucleus, with clear negative effects on FOXO1-dependent downstream pathways [60]. Further deregulation of FOXO1 was recently attributed, in the case of endometriosis, to NIMA-related kinase 2 (NEK2) overexpression, via NEK2-induced FOXO1 phosphorylation [61].

(F) Bone morphogenic protein 2 (BMP2), a member of the transforming growth factor β (TGFβ) family, is well known to be involved in decidualization [38]. BMP2 is primarily regulated by progesterone and participates in paracrine signaling between endometrial epithelial and stromal cells at the onset of decidualization [62]. Aberrant expression of BMP2 has been reported in cases of repeated implantation failure [63]. Recently, endometriosis was shown to be strongly associated with deregulation of TGFβ signaling, including key TGFβ regulators like SMAD4. Interestingly, BMP signaling was also shown to be impaired - a fact significantly reversed by exogenous BMP2 [64].

Table 2 summarizes the main molecular features of progesterone resistance. 

**Table 2:** Main molecular landmarks of progesterone resistance ARID1A, AT-rich interaction domain 1A; Ihh, Indian Hedgehog; HAND2, Heart and Neural Crest Derivatives Expressed 2; MIG-6, Mitogen-Inducible Gene 6; NOTCH, Neurogenic Locus Notch Homolog; FOXO1, Forkhead Box Protein O1; Calpain-7, a member of the calpain family of calcium-dependent cysteine proteases; AKT, Protein Kinase B; NEK2, NIMA-Related Kinase 2; TGFβ, Transforming Growth Factor Beta; BMP2, Bone Morphogenetic Protein 2

Progesterone resistance (main molecular landmarks)
Significant decrease in ARID1A expression
Down-regulation of the Ihh-pathway
Down-regulation of the HAND2 pathway
Down-regulation of the MIG-6
Down-regulated NOTCH signaling leading to reduced FOXO-1 expression
Elevated expression of Calpain-7 and downstream effect on AKT and FOXO-1
Elevated expression of NEK-2
Deregulation of TGFβ signalling
Deregulation of BMP2

Impact of endometriosis-associated inflammation on endometrial receptivity

Endometrial receptivity is established via decidualization. Decidualization is a progesterone-dependent transformation of the endometrial stroma, along with changes in the morphology of the endometrial epithelium. A key element of decidualization is the induction of a low-intensity, aseptic inflammation, which facilitates local immunomodulation toward maternal-fetal tolerance. Initially, the local cytokine profile supports a transient Th1/Th17 immune profile, which shifts rapidly to the Th2/Treg immune profile, being favorable for successful implantation. The main aim of decidualization is the priming of the endometrium to be receptive to the blastocyst in a coordinated fashion: maximal receptivity must be synchronized with the apposed blastocyst. Any molecular event interfering with the decidua-blastocyst synchronization contributes to implantation failure.

Endometriosis per se is considered an inflammatory condition. The inflammatory profile of endometriosis has been thoroughly investigated in several key anatomical compartments. The first site of interest was the peritoneal fluid, since it collects inflammatory cytokines directly from the peritoneal endometriotic implants. Several inflammatory cytokines, including tumor necrosis factor-alpha (TNF-α), interleukin-1 (IL-1), IL-6, IL-8, IL-17, and monocyte chemoattractant protein-1 (MCP-1), have been identified at significantly increased levels compared to healthy controls [65-67]. Furthermore, in the case of endometriosis, peritoneal fluid was suggested to have a direct negative impact on endometrial receptivity by altering the expression of leukemia-induced factor (LIF) and the integrin αvβ3 [68]. Interestingly, the endometrial fluid of women with endometriosis presented a pro-inflammatory profile, further supporting the thesis that endometriosis induces inflammation both in the ectopic and the eutopic endometrium [69]. This was in line with a previous report showing that endometrial tissue samples of women with endometriosis present a significant overexpression of Th1 cytokines, namely IL-1, IL-8, and TNF-α [70]. 

IL-1β is an important pro-inflammatory cytokine reported to be upregulated in the peritoneal fluid and peripheral blood of women with endometriosis [71]. IL-1β seems to disrupt both decidualization and implantation. It has been shown that IL-1β inhibits the decidualization of endometrial stromal cells by downregulating connexin 43, IGFBP-1, and PRL; this is achieved via the ERK and MAPK pathways [72]. Of note is the fact that connexin 43 is a gap junction protein involved in the intercellular transport of small metabolites among endometrial cells; downregulation of Cx43 seems to contribute to decidualization derangement [73].

In the same direction, IL-6 has been described as elevated in cases of endometriosis, both in the peritoneal and endometrial fluid [69,74]. IL-6 is normally involved in several processes during implantation and early fetal development [75]. Its signaling is achieved via the signal transducer and activator of transcription 3 (STAT3) and the MAPK pathways. Elevated IL-6 concentrations have been linked to infertility [76]. LIF, a member of the IL-6 family, is also altered and downregulated in the eutopic endometrium of women with endometriosis [77]. LIF signaling involves its receptor and gp130. LIF is a strong marker of implantation, being expressed mainly during the WOI [78]. 

Dysfunctional endometrium has also been attributed to elevated levels of IL-7. It has been shown that elevated endometrial IL-7 is associated with early pregnancy loss [79]. IL-7 signaling is considered to be involved in pregnancy loss by inducing Th17 immunity, the latter not favoring pregnancy maintenance [80]. Interestingly, IL-7 was found to be significantly elevated in the eutopic endometrium of women with endometriosis [81].

Impaired decidualization is a prevalent problem in endometriosis patients, who commonly have low levels of IL-11 in their eutopic endometrium [82]. IL-11 plays an important part in decidualization in both mouse and human systems, with regulation varying by species [83]. In mice, IL-11 expression increases after implantation, but in humans, it increases during the secretory phase of the menstrual cycle [84]. Evidence from in vitro data has shown that endometrial decidualized stromal cells derived from women with endometriosis present aberrant expression of IL-11, which is most likely caused by progesterone resistance and altered protein kinase A (PKA) signaling [83]. IL-11 down-regulation may lead to endometriosis-associated infertility via a negative impact on decidualization, implantation, and placentation. Proposed mechanisms involve altered natural killer cell differentiation [85] and decreased decidual cell survival [86].

Finally, an important inducer of the inflammatory environment in the eutopic endometrium of women with endometriosis is IL-17, most commonly produced by Th17 helper cells. IL-17 induces COX-2 and, thereafter, the production of prostaglandins. At the same time, IL-17 induces IL-8, which, in turn, upregulates the AKT pathway, a crucial inhibitor of decidualization [87]. Interestingly, therapeutic interventions against endometriosis seem to downregulate IL-17 [88]. All main contributors to defective decidualization are summarized in Table 3. 

**Table 3:** Main molecular contributors of defective decidualization in endometriosis LIF, Leukemia Inhibitory Factor; αvβ3, Integrin Alpha-v Beta-3; IL, Interleukin; TNFα, Tumor Necrosis Factor Alpha; IGFBP-1, Insulin-like Growth Factor Binding Protein-1

Defective decidualization (main molecular contributors)
Down-regulation of LIF
Down-regulation of αvβ3
Overexpression of IL-1, IL-8 and TNFα
IL-1β-induced down-regulation of Connexin-43, IGFBP-1 and prolactin
Overexpression of IL-6
Overexpression of IL-7
Reduced expression of IL-11
Overexpression of IL-17

Immunological profile of the eutopic endometrium in endometriosis

Macrophages

In normal endometrium, macrophages increase in number and peak immediately prior to menstruation, most likely in clearing endometrial apoptotic cells [89-91]. In normal endometrium, the main macrophage profile is M2, an anti-inflammatory cell population that is featured by IL-10 secretion, which in turn activates Th2 cells [92,93]. In the same direction, via chemokine (C-C motif) ligand 1 (CCL-1) production, a subtype of M2 cells has been reported to trigger regulatory T-cell recruitment, contributing to local immune suppression in favor of implantation [94,95]. The eutopic endometrium, in cases of endometriosis, is characterized by lower M2 populations throughout the menstrual cycle, with a concurrent development of the M1 population [96]. This macrophage profile is further enhanced by the elevated concentrations of MCP-1 and MIF [97,98], both strongly associated with inflammation. It has been shown that macrophages contribute to increased nerve fiber density, a fact linked to symptoms related to endometriosis [99]. Although this has not been clearly shown in the eutopic endometrium in the case of endometriosis, it could be considered a potential alteration towards implantation failure.

Uterine Natural Killer (uNK) Cells

uNK cells are the most abundant population of immune cells in the human endometrium, comprising up to 30% of the immune cells in the proliferative phase and 70% in the secretory phase [100,101]. This upregulation can be explained either by the effect of the cyclic changes in the endometrial stroma or by the immediate effect of steroid sex hormones. Normal uNK cells present with a unique immune phenotype, namely CD56⁺ CD16⁻ [91]. The downregulation of CD16 is associated with a decreased ability to secrete pro-inflammatory cytokines, along with reduced uNK cytotoxicity [102]. uNK cytotoxicity is regulated positively by NKp30 and NKp46 receptors [103,104], and negatively by killer cell immunoglobulin-like receptors (KIRs) [105]. In normal endometrium, uNK cytotoxicity appears to be reduced. Thus, during the peri-implantation period, uNK cells are abundant but less cytotoxic. Despite their reduced cytotoxicity, uNK cells are considered guardians against microbial infections while, at the same time, secreting vascular endothelial growth factor-C (VEGF-C), placental growth factor (PGF), and angiopoietin-2 (Ang-2), thereby inducing local angiogenesis to support early implantation [106]. uNK cells are also important in regulating trophoblast invasion by secreting an array of cytokines and chemokines, among which are LIF, granulocyte-colony stimulating factor (G-CSF), and TGF-β [102].

The alterations of uNK cells in the eutopic endometrium of women with endometriosis have not yet been fully clarified. A recent study, based both on the analysis of transcriptome data series and on validation experiments, has shown that the uNK population is significantly upregulated in the eutopic endometrium of women with endometriosis compared to controls [107]. It was also reported that activated uNK cells were upregulated in the case of endometriosis [107]. This was in line with a recent transcriptome meta-analysis reporting a higher number of uNK cells as a differential phenotypic presentation between endometriosis stages I-II and III-IV [108]. It was suggested that early-stage endometriosis is characterized by a more inflammatory eutopic endometrium, while advanced-stage endometriosis presents with less inflammation, as far as the eutopic endometrium is concerned - most possibly as a result of an M2 response to chronic stress. Interestingly, CD16⁺ uNK cells have been found in the eutopic endometrium of infertile women with endometriosis (Table 4), suggesting a potential cytotoxic response to trophoblast invasion and, thereafter, to implantation failure [109]. 

**Table 4:** Immunological profile of the eutopic endometrium uNK, uterine Natural Killer cells; CD, cluster of differentiation; Th, T-helper

Immunological profile of the eutopic endometrium (in terms of cellular populations)
Macrophages
Decrease in M2 populations
Increase in M1 populations
Uterine Natural Killer cells
Increase in uNK population
Elevated CD16-expressing uNK population
T-cells
Increase in CD8 populations
Increase in CD4/Th1 populations
Decrease in CD4/Th2 populations

T-cells

The role of T-cells is focused on antigen presentation and modulation of innate immunity. All T-cells express the receptor CD3. Additionally, T-cells express either CD8 or CD4 receptors, which classify them into the corresponding categories. Further cytokine profiling has categorized CD4 cells as Th1 (helper cells), Th2 (helper cells), Th17 (helper cells), follicular T helper cells, and T regulatory cells [91]. All T-cell categories present cyclical alterations in population size, being elevated during the proliferative phase.

CD8: CD8 cells - also called cytotoxic T-cells - act as destroyers of (a) cells infected by an external pathogen or (b) tumor cells. In the normal eutopic endometrium, CD8 cells are localized in the stroma and, less often, within the epithelium. Their activation is MHC-I mediated, a process that depends on antigen-presenting cells (APCs) exposed to a local threat. As a result, CD8 cells may secrete cytotoxic cytokines - like TNF-alpha - or cytotoxins - like perforin - while additionally inducing apoptosis via the Fas-FasL pathway [91]. CD8 cells are normally reduced during the secretory phase of the menstrual cycle, in the context of local immune tolerance, which contributes to successful implantation. Recent evidence has shown that CD8 cells are significantly more abundant in the eutopic endometrium of women with endometriosis, while, at the same time, the naïve CD8 population is reduced [107]. This finding implies a role for activated CD8 cells as contributors to local inflammation that may hinder successful implantation.

CD4-Th1 & Th2 cells: In the normal eutopic endometrium, the Th1 sub-group is the pro-inflammatory mediator. Being abundant during the proliferative phase [110], Th1 cells secrete an array of pro-inflammatory cytokines, among which IL-2 and interferon (IFN)-gamma are of great importance, since they have been reported as mediators of an unfriendly environment for the embryo to implant [111]. On the contrary, Th2 cells secrete anti-inflammatory cytokines, namely IL-6 and IL-10, which have been reported as supporters of early implantation [111]. Being more abundant in the secretory phase [112], Th2 cells inhibit the Th1 population, establishing a dynamically regulated equilibrium - from the Th1-dominant proliferative phase to the Th2-dominant secretory phase [91]. Unfortunately, evidence is rather weak at the moment regarding any clear-cut changes in the eutopic endometrium of women with endometriosis. A trend, however, has been noted recently, showing an elevation of Th1 and a decrease in Th2 populations, along with an upregulation of follicular T helper cells [107].

T-regulatory cells: T-regulatory cells are now considered the key regulators of endometrial biology. They interact with and modulate all other immune populations, along with their functions [113]. T-regulatory cell alterations have been associated with implantation failures [114]. Normally, T-regulatory cells increase in population size during the proliferative phase and are maintained up to the end of the secretory phase [115,116]. Since they are considered essential in establishing local immune tolerance, any alteration in their number or activity, in the case of endometriosis, could be associated with local immune dysfunction and a hostile decidua. The current evidence is not strong, since different methods have been applied to identify T-regulatory cells, leading to conflicting results. Of note is a recent meta-analysis showing the T-regulatory cell population to be unaltered in the case of endometriosis [107].

Epigenetic regulation of eutopic endometrium

Epigenetics involves all inherited alterations in gene expression through mechanisms other than changes to the DNA sequence. In that context, epigenetics refers to DNA modifications and chromosomal remodeling. DNA modifications are usually achieved via methylation at specific DNA bases, while chromosomal remodeling occurs through methylation or acetylation of histones, thereby affecting nucleosome dynamics.

Epigenetic regulation appears to be altered in the eutopic endometrium of women with endometriosis. Several studies have assessed the methylome of the eutopic endometrium in these women, compared to normal endometrium, and have concluded that a significant number of genes are modified in terms of methylation or acetylation [117].

Several alterations have been reported as relevant to eutopic endometrial physiology (Table 5): (A) Elevated DNA methylation of PR-B during the secretory phase is directly associated with decreased PR-B expression levels, and subsequently, with progesterone resistance [43]. (B) Local inflammation-induced hypoxia leads to the stabilization of enhancer of zeste homolog 2 (EZH2) and the upregulation of histone H3 trimethylated at lysine 27 (H3K27me3). This, in turn, negatively impacts IGFBP-1 expression, a key molecule in decidualization [118]. (C) Reduced levels of mixed lineage leukemia 1 (MLL1) and H3K4me3 in the eutopic endometrium may disturb COX gene expression, contributing to defective decidualization [119]. (D) Lower expression of histone deacetylase 3 (HDAC3) may downregulate genes associated with decidualization [120]. (E) Upregulation of the histone modifier SIRT1 in both the endometrial epithelium and stroma may suppress decidualization by reducing IGFBP-1 and PRL expression [121,122]. (F) Downregulation of homeobox A10 (HOXA10) expression is attributed to hypermethylation, caused by increased expression of DNA methyltransferase 3A [123,124], elevated H3K9me3, and reduced H3K9ac [125]. A recent systematic review highlighted that HOXA10 promoter methylation is commonly seen in the eutopic endometrium of women with endometriosis [126]. (G) Reduced activity of the p300/CREB-binding protein-associated factor (PCAF) decreases acetylation of HOXA10-regulated genes, further disrupting decidualization [127].

**Table 5:** Epigenetic alterations of the eutopic endometrium in case of endometriosis

Epigenetic alterations of the eutopic endometrium
Elevated DNA methylation of the progesterone receptor B
Stabilization of enhancer of zeste homolog 2 (EZH2) and the up-regulation of histone H3 trimethylated at lysine 27 (H3K27me3) levels
Reduced mixed lineage leukemia 1 (MLL1) and H3 trimethylated at lysine 4 (H3K4me3) levels
Lower histone deacetylase 3 (HDAC-3)
Up-regulation of the histone modifier SIRT1 in both endometrial epithelium and stroma
Down regulation of Homeobox A10 (HOXA-10) expression due to hypermethylation attributed to increased expression of DNA 3A methyltransferase
Reduced activity of the p300/CREB-binding protein-associated factor (PCAF)
Reduced ten-eleven translocation (TET) expression in the mid-secretory phase

In parallel, major enzymes like HDACs have been studied in terms of their overexpression or inhibition, showing notable effects on the transcriptional activity of target genes and levels of histone acetylation [120,128]. Any deviation from the normal epigenetic profile appears to affect eutopic endometrial function, ultimately leading to impaired implantation.

An emerging role for a new group of epigenetic enzymes, the ten-eleven translocation (TET) proteins, has also been demonstrated [129]. Involved in DNA demethylation and thus gene activation, TET expression was found to be reduced in the mid-secretory phase in women with endometriosis and infertility, suggesting a potential role for TET enzymes in implantation failure [130].

Role of endometriosis-related stress in the eutopic endometrium

Considering endometriosis to be a chronic inflammatory condition, it could be reasonably assumed that the eutopic endometrium is under chronic stress. It has been previously shown that corticotropin-releasing hormone (CRH), the main regulator of stress, regulates implantation by inducing apoptosis of locally activated T-cells, thus contributing to maternal immune tolerance [131]. Interestingly, it was later reported that the expression of CRH and urocortin was altered in both the eutopic endometrium and endometriotic foci [132,133]. Additionally, CRH receptor 1 was found to be upregulated in the eutopic endometrium in cases of endometriosis, which may further impair endometrial receptivity by deregulating the expression of galectin-1 - a key glycoprotein involved in receptivity and implantation [133,134].

## Conclusions

The current evidence supports the thesis that the eutopic endometrium presents significant alterations in the case of endometriosis. The evidence has been produced by observational studies aiming to highlight an association between changes in the eutopic endometrium and functional derangement. However, the clinical implications of these changes on contemporary practice have not yet been clarified. Properly designed studies are needed to determine whether such findings can be used to develop therapeutic interventions for endometriosis-related infertility.
